# Cholest-5-en-3β-yl *N*-phenyl­carbamate

**DOI:** 10.1107/S1600536809049010

**Published:** 2009-11-28

**Authors:** Mohsen Graia, Ghalib Raza Murad, Mehrzia Krimi Ammar, Sayed Hasan Mehdi, Rokiah Hashim

**Affiliations:** a Laboratoire de Matériaux et de Cristallochimie, Faculté des Sciences de Tunis, Université de Tunis–El Manar, 2092 El Manar II Tunis, Tunisia; b School of Industrial Technology, Universiti Sains Malaysia, 11800 Pulau Pinang, Malaysia; c Unité de Recherche de Chimie des Matériaux, ISSBAT, Université Tunis–Al Manar, 9 Rue Docteur Zouheir Safi, 1006 Tunis, Tunisia

## Abstract

In the title compound, C_34_H_51_NO_2_, the dihedral angle between the planes of the phenyl ring and the carbonyl group is 9.30 (2)°. No significant inter­molecular inter­actions are observed in the crystal structure. The C_5_H_11_ fragment is disordered over two positions with site occupancies of 0.611 (6) and 0.389 (6).

## Related literature

Cholesterol esterase is responsible for the hydrolysis of dietary cholesterol esters, fat-soluble vitamin esters, phospho­lipids and triacyl­glycerols, see: Chiou *et al.* (2008 ▶). Compounds containing a carbamate functionality are characterized as good inibitors of cholesterol esterase, see: Hosie *et al.* (1987 ▶). For comparative C—N bond lengths, see: Haramura *et al.* (2003 ▶); Hökelek & Ergün (2008 ▶).
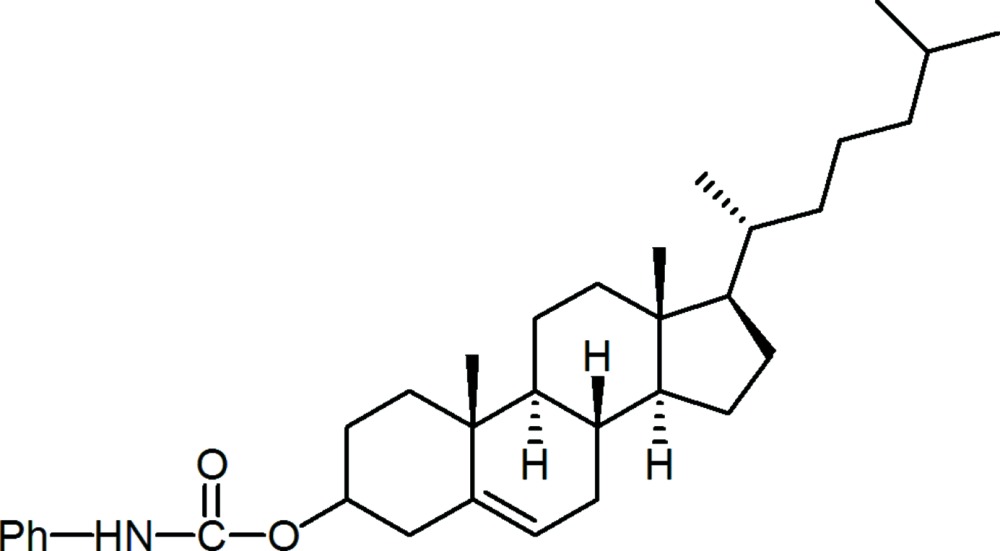


## Experimental

### 

#### Crystal data

C_34_H_51_NO_2_
*M* *_r_* = 505.75
Triclinic, 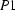

*a* = 6.330 (5) Å
*b* = 10.419 (5) Å
*c* = 12.028 (5) Å
α = 82.922 (5)°
β = 89.137 (5)°
γ = 73.141 (5)°
*V* = 753.2 (8) Å^3^
*Z* = 1
Mo *K*α radiation
μ = 0.07 mm^−1^
*T* = 293 K
0.37 × 0.32 × 0.11 mm

#### Data collection

Bruker SMART CCD area-detector diffractometer
Absorption correction: multi-scan (Coppens *et al.*, 1965 ▶) *T*_min_ = 0.955, *T*_max_ = 0.976
5215 measured reflections
3264 independent reflections
2578 reflections with *I* > 2σ(*I*)
*R*_int_ = 0.020

#### Refinement

*R*[*F* ^2^ > 2σ(*F* ^2^)] = 0.055
*wR*(*F* ^2^) = 0.152
*S* = 1.06
3264 reflections
361 parameters
13 restraints
H-atom parameters constrained
Δρ_max_ = 0.25 e Å^−3^
Δρ_min_ = −0.22 e Å^−3^

### 

Data collection: *SMART* (Bruker, 1998 ▶); cell refinement: *SAINT-Plus* (Bruker, 1998 ▶); data reduction: *SAINT-Plus* program(s) used to solve structure: *SHELXS97* (Sheldrick, 2008 ▶); program(s) used to refine structure: *SHELXL97* (Sheldrick, 2008 ▶); molecular graphics: *DIAMOND* (Brandenberg, 1999 ▶); software used to prepare material for publication: *WinGX* (Farrugia, 1999 ▶).

## Supplementary Material

Crystal structure: contains datablocks I, global. DOI: 10.1107/S1600536809049010/zq2013sup1.cif

Structure factors: contains datablocks I. DOI: 10.1107/S1600536809049010/zq2013Isup2.hkl

Additional supplementary materials:  crystallographic information; 3D view; checkCIF report

**Fig. 3.**
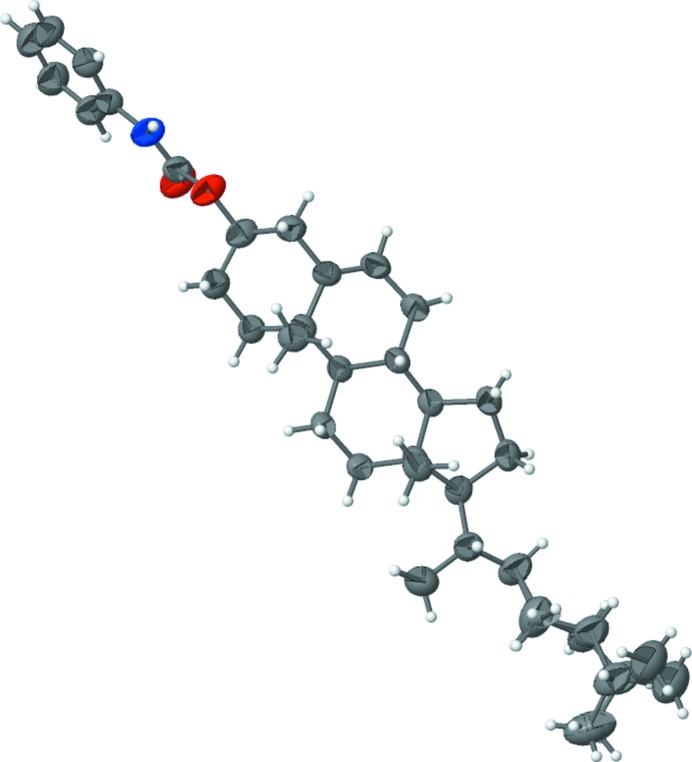
Molecular view of the title compound.

Enhanced figure: interactive version of Fig. 3

## Figures and Tables

**Fig. 1.**
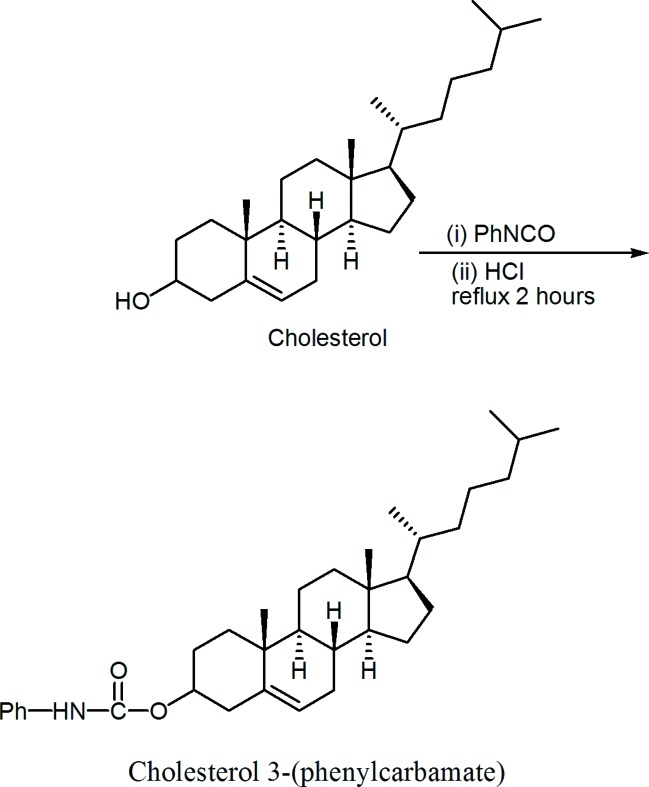
Chemical pathway of the formation of the cholesterol 3-(phenylcarbamate).

**Fig. 2.**
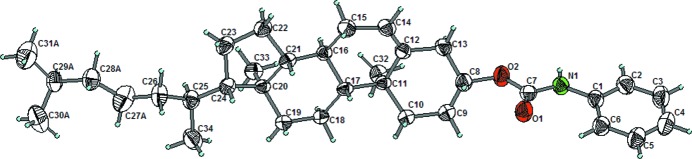
Molecular view of the title compound, showing the atom-numbering scheme. Displacement ellipsoids are drawn at 35% probabilit level.

## supplementary crystallographic information

### Comment

Cholesterol esterase is responsible for the hydrolysis of dietary cholesterol esters fat soluble vitamin ester, phospholipids and triacylglycerols (Chiou *et al.*, 2008). Compounds containing a carbamate functionality are characterized as good inibitors of cholesterol esterase (Hosie *et al.*, 1987). The molecular structure of (I) is shown in Fig. 2. The observed values for C_Ar_—C_Ar_ [1.379 (6) Å], C_sp3_—C_sp3_ [1.530 (9) Å], C_sp3_—O [1.463 (4) Å] and C_sp2_—O [1.204 (4) Å], are in the expected ranges. The C—N average distance [1.380 (4) Å] are well within the range found for C_8_H_15_N_3_O_7_ and C_16_H_12_N_2_O_2_ (Haramura *et al.*, 2003; Hökelek *et al.*, 2008). The dihedral angle between the planes of the phenyl and carbonyl group is 9.30(0.23)°. In the crystal structure, no significant intermolecular interactions are observed.

### Experimental

A mixture of cholesterol (1.93 g m 5 mmol) and phenylisocyanate (0.60 ml, 5 mmol) were taken in 40 ml of chloroform. Catalytic amount of HCl was added to it. The reaction mixture was refluxed on water bath for two hours then distilled under reduced pressure. The crude product thus obtained was crystallized from acetone - petroleum ether (9:1) mixture to afford the compound cholesterol 3-(phenylcarbamate) (Fig.1) as shining crystals (1.50 g m), melting point 160 0 C.

### Refinement

The structure was solved by direct methods with *SHELX97* program, and refined anisotropically by the full-matrix least-squares methods for all non-H atoms. The positions of the H atoms were placed at geometrically idealized positions (C–H = 0.96 Å, N–H = 0.86 Å) and constrained to ride on their parent atoms with *U*_iso_(H) = 1.2*U*_eq_(carrier atom). The disordered model was refined by using the tools available in *SHELXL97* (Sheldrick, 2008). In the absence of significant anomalous dispersion effects, Friedel pairs were merged.

### Crystal data

**Table:**

C_34_H_51_NO_2_	*Z* = 1
*M**_r_* = 505.75	*F*(000) = 278
Triclinic, *P*1	*D*_x_ = 1.115 Mg m^−^^3^
Hall symbol: P 1	Melting point: 433 K
*a* = 6.330 (5) Å	Mo *K*α radiation, λ = 0.71073 Å
*b* = 10.419 (5) Å	θ = 4.5–27.0°
*c* = 12.028 (5) Å	µ = 0.07 mm^−^^1^
α = 82.922 (5)°	*T* = 293 K
β = 89.137 (5)°	Prism, colourless
γ = 73.141 (5)°	0.37 × 0.32 × 0.11 mm
*V* = 753.2 (8) Å^3^	

### Data collection

**Table:**

Bruker SMART CCD area-detector diffractometer	3264 independent reflections
Radiation source: sealed tube	2578 reflections with *I* > 2σ(*I*)
graphite	*R*_int_ = 0.020
φ and ω scans	θ_max_ = 27.0°, θ_min_ = 4.5°
Absorption correction: multi-scan (Coppens *et al.*, 1965)	*h* = −8→8
*T*_min_ = 0.955, *T*_max_ = 0.976	*k* = −13→13
5215 measured reflections	*l* = −15→15

### Refinement

**Table:**

Refinement on *F*^2^	Secondary atom site location: difference Fourier map
Least-squares matrix: full	Hydrogen site location: inferred from neighbouring sites
*R*[*F*^2^ > 2σ(*F*^2^)] = 0.055	H-atom parameters constrained
*wR*(*F*^2^) = 0.152	*w* = 1/[σ^2^(*F*_o_^2^) + (0.1011*P*)^2^] where *P* = (*F*_o_^2^ + 2*F*_c_^2^)/3
*S* = 1.06	(Δ/σ)_max_ < 0.001
3264 reflections	Δρ_max_ = 0.25 e Å^−^^3^
361 parameters	Δρ_min_ = −0.21 e Å^−^^3^
13 restraints	Extinction correction: *SHELXL97* (Sheldrick, 2008), Fc^*^=kFc[1+0.001xFc^2^λ^3^/sin(2θ)]^-1/4^
Primary atom site location: structure-invariant direct methods	Extinction coefficient: 0.23 (3)

### Special details

**Table:**

Geometry. All e.s.d.'s (except the e.s.d. in the dihedral angle between two l.s. planes) are estimated using the full covariance matrix. The cell e.s.d.'s are taken into account individually in the estimation of e.s.d.'s in distances, angles and torsion angles; correlations between e.s.d.'s in cell parameters are only used when they are defined by crystal symmetry. An approximate (isotropic) treatment of cell e.s.d.'s is used for estimating e.s.d.'s involving l.s. planes.
Refinement. Refinement of *F*^2^ against ALL reflections. The weighted *R*-factor *wR* and goodness of fit *S* are based on *F*^2^, conventional *R*-factors *R* are based on *F*, with *F* set to zero for negative *F*^2^. The threshold expression of *F*^2^ > σ(*F*^2^) is used only for calculating *R*-factors(gt) *etc*. and is not relevant to the choice of reflections for refinement. *R*-factors based on *F*^2^ are statistically about twice as large as those based on *F*, and *R*- factors based on ALL data will be even larger.

### Fractional atomic coordinates and isotropic or equivalent isotropic displacement parameters (Å^2^)

**Table:**

	*x*	*y*	*z*	*U*_iso_*/*U*_eq_	Occ. (<1)
O2	0.2523 (4)	0.0541 (2)	0.1416 (2)	0.0718 (7)	
O1	−0.0134 (5)	−0.0218 (3)	0.2290 (2)	0.0774 (7)	
N1	0.1371 (5)	−0.0948 (3)	0.0663 (3)	0.0663 (7)	
H7	0.2406	−0.0883	0.0206	0.080*	
C1	0.0159 (5)	−0.1820 (3)	0.0423 (3)	0.0572 (7)	
C2	0.0865 (7)	−0.2584 (4)	−0.0441 (3)	0.0719 (9)	
H2	0.2103	−0.2508	−0.0839	0.086*	
C3	−0.0254 (8)	−0.3463 (4)	−0.0720 (3)	0.0836 (12)	
H3	0.0222	−0.3970	−0.1308	0.100*	
C4	−0.2075 (8)	−0.3587 (4)	−0.0124 (4)	0.0855 (12)	
H4	−0.2824	−0.4184	−0.0306	0.103*	
C5	−0.2777 (7)	−0.2832 (4)	0.0732 (3)	0.0780 (10)	
H5	−0.4014	−0.2914	0.1129	0.094*	
C6	−0.1677 (6)	−0.1945 (3)	0.1019 (3)	0.0669 (8)	
H6	−0.2166	−0.1438	0.1607	0.080*	
C7	0.1109 (5)	−0.0195 (3)	0.1525 (3)	0.0575 (7)	
C8	0.2412 (6)	0.1471 (3)	0.2247 (3)	0.0614 (8)	
H8	0.1846	0.1127	0.2949	0.074*	
C9	0.0919 (6)	0.2844 (4)	0.1824 (3)	0.0667 (8)	
H9A	−0.0575	0.2795	0.1735	0.080*	
H9B	0.1405	0.3153	0.1098	0.080*	
C10	0.0950 (5)	0.3847 (3)	0.2650 (3)	0.0598 (7)	
H10A	0.0325	0.3575	0.3349	0.072*	
H10B	0.0013	0.4733	0.2350	0.072*	
C11	0.3262 (4)	0.3957 (3)	0.2896 (2)	0.0467 (6)	
C12	0.4815 (5)	0.2536 (3)	0.3203 (2)	0.0508 (6)	
C13	0.4731 (5)	0.1510 (3)	0.2437 (3)	0.0603 (7)	
H13A	0.5356	0.1730	0.1723	0.072*	
H13B	0.5623	0.0622	0.2762	0.072*	
C14	0.6185 (5)	0.2199 (3)	0.4074 (3)	0.0606 (7)	
H14	0.7048	0.1303	0.4210	0.073*	
C15	0.6460 (6)	0.3144 (3)	0.4860 (3)	0.0620 (8)	
H15A	0.8023	0.2999	0.4996	0.074*	
H15B	0.5813	0.2935	0.5571	0.074*	
C16	0.5397 (4)	0.4628 (3)	0.4424 (2)	0.0470 (6)	
H16	0.6339	0.4908	0.3848	0.056*	
C17	0.3115 (4)	0.4793 (3)	0.3884 (2)	0.0458 (6)	
H17	0.2274	0.4420	0.4462	0.055*	
C18	0.1833 (5)	0.6304 (3)	0.3603 (3)	0.0542 (7)	
H18A	0.2513	0.6683	0.2967	0.065*	
H18B	0.0334	0.6373	0.3378	0.065*	
C19	0.1740 (5)	0.7154 (3)	0.4559 (3)	0.0526 (6)	
H19A	0.0894	0.6858	0.5165	0.063*	
H19B	0.0990	0.8093	0.4297	0.063*	
C20	0.4049 (4)	0.7038 (3)	0.5001 (2)	0.0457 (6)	
C21	0.5166 (5)	0.5505 (3)	0.5344 (2)	0.0482 (6)	
H21	0.4199	0.5203	0.5894	0.058*	
C22	0.7243 (5)	0.5459 (3)	0.5995 (3)	0.0624 (8)	
H22A	0.7714	0.4641	0.6521	0.075*	
H22B	0.8439	0.5500	0.5491	0.075*	
C23	0.6544 (6)	0.6705 (4)	0.6608 (3)	0.0659 (8)	
H23A	0.7596	0.7222	0.6485	0.079*	
H23B	0.6480	0.6440	0.7407	0.079*	
C24	0.4207 (5)	0.7575 (3)	0.6134 (2)	0.0529 (7)	
H24	0.3109	0.7322	0.6627	0.063*	
C25	0.3864 (6)	0.9096 (3)	0.6144 (3)	0.0625 (8)	
H25	0.4984	0.9344	0.5663	0.075*	
C32	0.4123 (6)	0.4624 (3)	0.1839 (3)	0.0598 (7)	
H32A	0.3008	0.5430	0.1541	0.072*	
H32B	0.4472	0.4006	0.1288	0.072*	
H32C	0.5426	0.4851	0.2031	0.072*	
C33	0.5342 (5)	0.7645 (3)	0.4101 (3)	0.0565 (7)	
H33A	0.6725	0.7648	0.4416	0.068*	
H33B	0.4501	0.8554	0.3835	0.068*	
H33C	0.5612	0.7112	0.3489	0.068*	
C34	0.1633 (9)	0.9962 (4)	0.5678 (4)	0.0953 (13)	
H34A	0.0494	0.9659	0.6065	0.114*	
H34B	0.1535	0.9890	0.4894	0.114*	
H34C	0.1452	1.0887	0.5780	0.114*	
C26	0.4242 (8)	0.9410 (4)	0.7323 (3)	0.0761 (10)	
H26A	0.2970	0.9367	0.7770	0.091*	
H26B	0.5513	0.8714	0.7666	0.091*	
C27A	0.460 (4)	1.0713 (18)	0.7362 (16)	0.101 (7)	0.611 (6)
H27A	0.3164	1.1383	0.7287	0.121*	0.611 (6)
H27B	0.5415	1.0892	0.6700	0.121*	0.611 (6)
C28A	0.576 (2)	1.0972 (12)	0.8337 (10)	0.085 (3)	0.611 (6)
H28A	0.7177	1.0286	0.8435	0.102*	0.611 (6)
H28B	0.4913	1.0841	0.8999	0.102*	0.611 (6)
C29A	0.6166 (15)	1.2323 (7)	0.8311 (6)	0.0817 (16)	0.611 (6)
H29A	0.6831	1.2502	0.7589	0.098*	0.611 (6)
C30A	0.410 (5)	1.347 (2)	0.838 (3)	0.119 (3)	0.611 (6)
H30A	0.3535	1.3418	0.9120	0.143*	0.611 (6)
H30B	0.3008	1.3407	0.7853	0.143*	0.611 (6)
H30C	0.4422	1.4314	0.8193	0.143*	0.611 (6)
C31A	0.783 (2)	1.2299 (12)	0.9221 (10)	0.130 (3)	0.611 (6)
H31A	0.7853	1.3206	0.9282	0.156*	0.611 (6)
H31B	0.9274	1.1771	0.9030	0.156*	0.611 (6)
H31C	0.7419	1.1906	0.9924	0.156*	0.611 (6)
C27B	0.476 (4)	1.0808 (18)	0.737 (2)	0.066 (6)	0.389 (6)
H27C	0.6123	1.0811	0.6995	0.079*	0.389 (6)
H27D	0.3570	1.1554	0.7013	0.079*	0.389 (6)
C28B	0.496 (4)	1.092 (2)	0.8641 (17)	0.085 (3)	0.389 (6)
H28C	0.3513	1.1051	0.8967	0.102*	0.389 (6)
H28D	0.5921	1.0076	0.9003	0.102*	0.389 (6)
C29B	0.587 (2)	1.2088 (12)	0.8899 (9)	0.0817 (16)	0.389 (6)
H29B	0.6020	1.2033	0.9714	0.098*	0.389 (6)
C30B	0.424 (7)	1.343 (4)	0.849 (6)	0.119 (3)	0.389 (6)
H30D	0.4433	1.4108	0.8909	0.143*	0.389 (6)
H30E	0.2763	1.3357	0.8577	0.143*	0.389 (6)
H30F	0.4474	1.3665	0.7707	0.143*	0.389 (6)
C31B	0.817 (3)	1.2017 (19)	0.8385 (16)	0.130 (3)	0.389 (6)
H31D	0.8109	1.1950	0.7598	0.156*	0.389 (6)
H31E	0.9259	1.1238	0.8748	0.156*	0.389 (6)
H31F	0.8557	1.2819	0.8493	0.156*	0.389 (6)

### Atomic displacement parameters (Å^2^)

**Table:**

	*U*^11^	*U*^22^	*U*^33^	*U*^12^	*U*^13^	*U*^23^
O2	0.0795 (17)	0.0731 (14)	0.0795 (15)	−0.0423 (13)	0.0218 (12)	−0.0269 (12)
O1	0.0785 (16)	0.0864 (17)	0.0836 (16)	−0.0433 (14)	0.0241 (13)	−0.0283 (13)
N1	0.0663 (17)	0.0631 (15)	0.0789 (17)	−0.0292 (13)	0.0182 (13)	−0.0213 (13)
C1	0.0658 (19)	0.0444 (14)	0.0601 (16)	−0.0154 (13)	−0.0076 (14)	−0.0021 (12)
C2	0.080 (2)	0.0641 (19)	0.070 (2)	−0.0171 (17)	−0.0068 (17)	−0.0099 (15)
C3	0.108 (3)	0.071 (2)	0.073 (2)	−0.024 (2)	−0.021 (2)	−0.0192 (17)
C4	0.106 (3)	0.073 (2)	0.088 (3)	−0.043 (2)	−0.026 (2)	−0.0040 (19)
C5	0.087 (3)	0.071 (2)	0.085 (2)	−0.0402 (19)	−0.0092 (19)	−0.0018 (18)
C6	0.074 (2)	0.0590 (17)	0.0726 (19)	−0.0268 (16)	0.0010 (16)	−0.0072 (15)
C7	0.0537 (16)	0.0477 (14)	0.0714 (19)	−0.0156 (13)	0.0018 (14)	−0.0064 (13)
C8	0.0650 (19)	0.0624 (17)	0.0655 (18)	−0.0292 (15)	0.0138 (15)	−0.0162 (14)
C9	0.0517 (17)	0.069 (2)	0.084 (2)	−0.0194 (16)	0.0026 (15)	−0.0239 (17)
C10	0.0440 (15)	0.0613 (17)	0.0761 (19)	−0.0137 (14)	−0.0013 (13)	−0.0193 (14)
C11	0.0404 (13)	0.0464 (13)	0.0523 (14)	−0.0131 (11)	0.0025 (11)	−0.0015 (11)
C12	0.0471 (14)	0.0446 (13)	0.0603 (15)	−0.0139 (11)	0.0069 (12)	−0.0038 (11)
C13	0.0587 (18)	0.0479 (15)	0.0745 (19)	−0.0152 (13)	0.0049 (14)	−0.0093 (13)
C14	0.0612 (18)	0.0431 (13)	0.0706 (18)	−0.0087 (12)	−0.0046 (14)	0.0035 (12)
C15	0.0665 (19)	0.0474 (15)	0.0658 (17)	−0.0106 (14)	−0.0158 (15)	0.0053 (13)
C16	0.0410 (13)	0.0446 (13)	0.0522 (14)	−0.0106 (11)	−0.0018 (11)	0.0023 (10)
C17	0.0374 (13)	0.0484 (13)	0.0523 (14)	−0.0152 (11)	0.0028 (11)	−0.0019 (11)
C18	0.0412 (14)	0.0517 (14)	0.0657 (16)	−0.0064 (12)	−0.0090 (12)	−0.0088 (12)
C19	0.0400 (14)	0.0542 (15)	0.0632 (16)	−0.0111 (12)	0.0010 (12)	−0.0118 (12)
C20	0.0391 (13)	0.0452 (13)	0.0520 (14)	−0.0126 (11)	0.0028 (11)	−0.0018 (11)
C21	0.0450 (14)	0.0492 (14)	0.0501 (14)	−0.0177 (12)	−0.0036 (11)	0.0053 (11)
C22	0.0599 (17)	0.0540 (15)	0.0712 (18)	−0.0173 (14)	−0.0187 (15)	0.0046 (13)
C23	0.0672 (19)	0.0666 (18)	0.0647 (17)	−0.0229 (16)	−0.0189 (14)	−0.0001 (14)
C24	0.0582 (17)	0.0545 (15)	0.0504 (14)	−0.0252 (13)	0.0016 (12)	−0.0018 (12)
C25	0.071 (2)	0.0567 (16)	0.0629 (17)	−0.0242 (15)	−0.0028 (15)	−0.0037 (13)
C32	0.0684 (18)	0.0551 (15)	0.0560 (15)	−0.0217 (14)	0.0056 (13)	0.0012 (13)
C33	0.0571 (17)	0.0515 (15)	0.0602 (16)	−0.0189 (13)	0.0036 (13)	0.0034 (12)
C34	0.102 (3)	0.069 (2)	0.104 (3)	−0.001 (2)	−0.023 (2)	−0.024 (2)
C26	0.106 (3)	0.066 (2)	0.0658 (19)	−0.040 (2)	−0.0018 (19)	−0.0076 (15)
C27A	0.132 (13)	0.097 (12)	0.085 (10)	−0.056 (9)	−0.020 (8)	0.005 (7)
C28A	0.112 (9)	0.066 (3)	0.085 (7)	−0.036 (5)	−0.006 (5)	−0.009 (4)
C29A	0.126 (5)	0.078 (3)	0.051 (3)	−0.041 (3)	0.011 (4)	−0.016 (3)
C30A	0.146 (7)	0.083 (3)	0.133 (9)	−0.030 (4)	0.023 (4)	−0.041 (4)
C31A	0.156 (8)	0.125 (6)	0.122 (7)	−0.058 (6)	−0.027 (7)	−0.022 (5)
C27B	0.098 (13)	0.031 (6)	0.079 (12)	−0.030 (7)	0.029 (9)	−0.023 (6)
C28B	0.112 (9)	0.066 (3)	0.085 (7)	−0.036 (5)	−0.006 (5)	−0.009 (4)
C29B	0.126 (5)	0.078 (3)	0.051 (3)	−0.041 (3)	0.011 (4)	−0.016 (3)
C30B	0.146 (7)	0.083 (3)	0.133 (9)	−0.030 (4)	0.023 (4)	−0.041 (4)
C31B	0.156 (8)	0.125 (6)	0.122 (7)	−0.058 (6)	−0.027 (7)	−0.022 (5)

### Geometric parameters (Å, °)

**Table:**

O2—C7	1.332 (4)	C22—C23	1.523 (5)
O2—C8	1.463 (4)	C22—H22A	0.9700
O1—C7	1.204 (4)	C22—H22B	0.9700
N1—C7	1.356 (4)	C23—C24	1.567 (5)
N1—C1	1.405 (4)	C23—H23A	0.9700
N1—H7	0.8600	C23—H23B	0.9700
C1—C2	1.377 (5)	C24—C25	1.537 (4)
C1—C6	1.386 (5)	C24—H24	0.9800
C2—C3	1.384 (6)	C25—C34	1.511 (6)
C2—H2	0.9300	C25—C26	1.532 (5)
C3—C4	1.377 (7)	C25—H25	0.9800
C3—H3	0.9300	C32—H32A	0.9600
C4—C5	1.364 (6)	C32—H32B	0.9600
C4—H4	0.9300	C32—H32C	0.9600
C5—C6	1.385 (5)	C33—H33A	0.9600
C5—H5	0.9300	C33—H33B	0.9600
C6—H6	0.9300	C33—H33C	0.9600
C8—C9	1.500 (5)	C34—H34A	0.9600
C8—C13	1.502 (5)	C34—H34B	0.9600
C8—H8	0.9800	C34—H34C	0.9600
C9—C10	1.532 (4)	C26—C27A	1.446 (19)
C9—H9A	0.9700	C26—C27B	1.59 (2)
C9—H9B	0.9700	C26—H26A	0.9700
C10—C11	1.536 (4)	C26—H26B	0.9700
C10—H10A	0.9700	C27A—C28A	1.49 (2)
C10—H10B	0.9700	C27A—H27A	0.9700
C11—C12	1.526 (4)	C27A—H27B	0.9700
C11—C32	1.541 (4)	C28A—C29A	1.499 (13)
C11—C17	1.544 (4)	C28A—H28A	0.9700
C12—C14	1.317 (4)	C28A—H28B	0.9700
C12—C13	1.508 (4)	C29A—C30A	1.50 (2)
C13—H13A	0.9700	C29A—C31A	1.525 (13)
C13—H13B	0.9700	C29A—H29A	0.9800
C14—C15	1.492 (5)	C30A—H30A	0.9600
C14—H14	0.9300	C30A—H30B	0.9600
C15—C16	1.525 (4)	C30A—H30C	0.9600
C15—H15A	0.9700	C31A—H31A	0.9600
C15—H15B	0.9700	C31A—H31B	0.9600
C16—C21	1.500 (4)	C31A—H31C	0.9600
C16—C17	1.547 (4)	C27B—C28B	1.56 (3)
C16—H16	0.9800	C27B—H27C	0.9700
C17—C18	1.545 (4)	C27B—H27D	0.9700
C17—H17	0.9800	C28B—C29B	1.55 (2)
C18—C19	1.527 (4)	C28B—H28C	0.9700
C18—H18A	0.9700	C28B—H28D	0.9700
C18—H18B	0.9700	C29B—C30B	1.50 (3)
C19—C20	1.529 (4)	C29B—C31B	1.56 (2)
C19—H19A	0.9700	C29B—H29B	0.9800
C19—H19B	0.9700	C30B—H30D	0.9600
C20—C33	1.532 (4)	C30B—H30E	0.9600
C20—C24	1.551 (4)	C30B—H30F	0.9600
C20—C21	1.553 (4)	C31B—H31D	0.9600
C21—C22	1.526 (4)	C31B—H31E	0.9600
C21—H21	0.9800	C31B—H31F	0.9600
			
C7—O2—C8	116.7 (2)	C20—C21—H21	106.0
C7—N1—C1	127.9 (3)	C23—C22—C21	104.1 (3)
C7—N1—H7	116.1	C23—C22—H22A	110.9
C1—N1—H7	116.1	C21—C22—H22A	110.9
C2—C1—C6	119.5 (3)	C23—C22—H22B	110.9
C2—C1—N1	117.5 (3)	C21—C22—H22B	110.9
C6—C1—N1	123.0 (3)	H22A—C22—H22B	109.0
C1—C2—C3	120.4 (4)	C22—C23—C24	107.4 (2)
C1—C2—H2	119.8	C22—C23—H23A	110.2
C3—C2—H2	119.8	C24—C23—H23A	110.2
C4—C3—C2	119.9 (4)	C22—C23—H23B	110.2
C4—C3—H3	120.0	C24—C23—H23B	110.2
C2—C3—H3	120.0	H23A—C23—H23B	108.5
C5—C4—C3	119.7 (4)	C25—C24—C20	119.0 (2)
C5—C4—H4	120.1	C25—C24—C23	112.1 (3)
C3—C4—H4	120.1	C20—C24—C23	103.1 (2)
C4—C5—C6	121.0 (4)	C25—C24—H24	107.4
C4—C5—H5	119.5	C20—C24—H24	107.4
C6—C5—H5	119.5	C23—C24—H24	107.4
C5—C6—C1	119.4 (3)	C34—C25—C26	110.4 (3)
C5—C6—H6	120.3	C34—C25—C24	113.3 (3)
C1—C6—H6	120.3	C26—C25—C24	111.0 (3)
O1—C7—O2	125.1 (3)	C34—C25—H25	107.3
O1—C7—N1	126.0 (3)	C26—C25—H25	107.3
O2—C7—N1	108.8 (3)	C24—C25—H25	107.3
O2—C8—C9	110.1 (3)	C11—C32—H32A	109.5
O2—C8—C13	106.7 (3)	C11—C32—H32B	109.5
C9—C8—C13	111.5 (3)	H32A—C32—H32B	109.5
O2—C8—H8	109.5	C11—C32—H32C	109.5
C9—C8—H8	109.5	H32A—C32—H32C	109.5
C13—C8—H8	109.5	H32B—C32—H32C	109.5
C8—C9—C10	110.1 (3)	C20—C33—H33A	109.5
C8—C9—H9A	109.6	C20—C33—H33B	109.5
C10—C9—H9A	109.6	H33A—C33—H33B	109.5
C8—C9—H9B	109.6	C20—C33—H33C	109.5
C10—C9—H9B	109.6	H33A—C33—H33C	109.5
H9A—C9—H9B	108.2	H33B—C33—H33C	109.5
C9—C10—C11	114.1 (2)	C25—C34—H34A	109.5
C9—C10—H10A	108.7	C25—C34—H34B	109.5
C11—C10—H10A	108.7	H34A—C34—H34B	109.5
C9—C10—H10B	108.7	C25—C34—H34C	109.5
C11—C10—H10B	108.7	H34A—C34—H34C	109.5
H10A—C10—H10B	107.6	H34B—C34—H34C	109.5
C12—C11—C10	108.6 (2)	C27A—C26—C25	114.6 (8)
C12—C11—C32	108.7 (2)	C27A—C26—C27B	2.7 (17)
C10—C11—C32	109.7 (3)	C25—C26—C27B	115.0 (9)
C12—C11—C17	110.2 (2)	C27A—C26—H26A	108.6
C10—C11—C17	108.5 (2)	C25—C26—H26A	108.6
C32—C11—C17	111.2 (2)	C27B—C26—H26A	110.6
C14—C12—C13	120.6 (3)	C27A—C26—H26B	108.6
C14—C12—C11	123.2 (3)	C25—C26—H26B	108.6
C13—C12—C11	116.1 (2)	C27B—C26—H26B	106.1
C8—C13—C12	111.8 (3)	H26A—C26—H26B	107.6
C8—C13—H13A	109.2	C26—C27A—C28A	120.6 (14)
C12—C13—H13A	109.2	C26—C27A—H27A	107.2
C8—C13—H13B	109.2	C28A—C27A—H27A	107.2
C12—C13—H13B	109.2	C26—C27A—H27B	107.2
H13A—C13—H13B	107.9	C28A—C27A—H27B	107.2
C12—C14—C15	125.0 (3)	H27A—C27A—H27B	106.8
C12—C14—H14	117.5	C27A—C28A—C29A	118.3 (11)
C15—C14—H14	117.5	C27A—C28A—H28A	107.7
C14—C15—C16	113.3 (2)	C29A—C28A—H28A	107.7
C14—C15—H15A	108.9	C27A—C28A—H28B	107.7
C16—C15—H15A	108.9	C29A—C28A—H28B	107.7
C14—C15—H15B	108.9	H28A—C28A—H28B	107.1
C16—C15—H15B	108.9	C28A—C29A—C30A	113.7 (15)
H15A—C15—H15B	107.7	C28A—C29A—C31A	111.1 (8)
C21—C16—C15	111.2 (2)	C30A—C29A—C31A	110.7 (16)
C21—C16—C17	110.5 (2)	C28A—C29A—H29A	107.0
C15—C16—C17	109.3 (2)	C30A—C29A—H29A	107.0
C21—C16—H16	108.6	C31A—C29A—H29A	107.0
C15—C16—H16	108.6	C28B—C27B—C26	105.2 (15)
C17—C16—H16	108.6	C28B—C27B—H27C	110.7
C11—C17—C18	114.2 (2)	C26—C27B—H27C	110.7
C11—C17—C16	112.8 (2)	C28B—C27B—H27D	110.7
C18—C17—C16	110.7 (2)	C26—C27B—H27D	110.7
C11—C17—H17	106.2	H27C—C27B—H27D	108.8
C18—C17—H17	106.2	C29B—C28B—C27B	114.6 (14)
C16—C17—H17	106.2	C29B—C28B—H28C	108.6
C19—C18—C17	114.8 (2)	C27B—C28B—H28C	108.6
C19—C18—H18A	108.6	C29B—C28B—H28D	108.6
C17—C18—H18A	108.6	C27B—C28B—H28D	108.6
C19—C18—H18B	108.6	H28C—C28B—H28D	107.6
C17—C18—H18B	108.6	C30B—C29B—C28B	110 (2)
H18A—C18—H18B	107.5	C30B—C29B—C31B	110 (2)
C18—C19—C20	111.6 (2)	C28B—C29B—C31B	113.5 (11)
C18—C19—H19A	109.3	C30B—C29B—H29B	108.0
C20—C19—H19A	109.3	C28B—C29B—H29B	108.0
C18—C19—H19B	109.3	C31B—C29B—H29B	108.0
C20—C19—H19B	109.3	C29B—C30B—H30D	109.5
H19A—C19—H19B	108.0	C29B—C30B—H30E	109.5
C19—C20—C33	110.6 (2)	H30D—C30B—H30E	109.5
C19—C20—C24	117.2 (2)	C29B—C30B—H30F	109.5
C33—C20—C24	110.7 (2)	H30D—C30B—H30F	109.5
C19—C20—C21	105.9 (2)	H30E—C30B—H30F	109.5
C33—C20—C21	111.9 (2)	C29B—C31B—H31D	109.5
C24—C20—C21	99.9 (2)	C29B—C31B—H31E	109.5
C16—C21—C22	118.3 (2)	H31D—C31B—H31E	109.5
C16—C21—C20	115.7 (2)	C29B—C31B—H31F	109.5
C22—C21—C20	103.9 (2)	H31D—C31B—H31F	109.5
C16—C21—H21	106.0	H31E—C31B—H31F	109.5
C22—C21—H21	106.0		
